# Factors associated with premature birth: a case-control study

**DOI:** 10.1590/1984-0462/2022/40/2020486IN

**Published:** 2022-05-06

**Authors:** Érica Cesário Defilipo, Paula Silva de Carvalho Chagas, Carolyne de Miranda Drumond, Luiz Cláudio Ribeiro

**Affiliations:** a Universidade Federal de Juiz de Fora. Governador Valadares, MG, Brazil.; b Universidade Federal de Juiz de Fora. Juiz de Fora, MG, Brazil.

**Keywords:** Infant, premature, Prenatal care, Risk factors, Pregnancy complications, Violence against women, Recém-nascido prematuro, Cuidado pré-natal, Fatores de risco, Complicações na gravidez, Violência contra a mulher

## Abstract

**Objective:**

To analyze the socioeconomic, demographic, environmental, reproductive, behavioral, and health-care factors associated with preterm birth.

**Methods:**

Case-control study, with case group composed of preterm infants and the control group by full term live births. Each case was paired with two controls according to sex and date of birth. Interviews were carried out with the mothers, as well as analysis of medical records. A logistic regression model was used for data analysis following the hierarchical order of entry of the blocks.

**Results:**

221 live births were allocated in the case group and 442 in the control group. After analysis adjusted for other factors under study, the highest chances of prematurity were associated with being the first child (OR 1.96; 95%CI 1.34–2.86; p=0.001); mothers with the highest income (OR 2.08; 95%CI 1.41–3.08; p<0.001), mothers with previous preterm births (OR 3.98; 95%CI 2.04–7.79; p<0.001), mothers that suffered violence during pregnancy (OR 2.50; 95%CI 1.31–4.78; p=0.005) and underwent cesarean section (OR 2.35; 95%CI 1.63–3.38; p<0.001). Live births to mothers who had more than six prenatal consultations had a lower risk of prematurity (OR 0.39; 95%CI 0.26–0.58; p<0.001).

**Conclusions:**

The factors associated with a higher chance of prematurity were: higher family income, previous preterm child, primiparity, violence against pregnant women and cesarean section. Having attended more than six prenatal visits was associated with a lower chance of premature birth. Violence against pregnant women showed a strong and consistent association, remaining in all final models, and should serve as an alert for the population and professionals.

## RESUMO

**Objetivo:**

Analisar os fatores socioeconômicos, demográficos, ambientais, reprodutivos, comportamentais e de assistência à saúde associados à prematuridade.

**Métodos:**

Estudo caso-controle, sendo o grupo caso composto de prematuros e o grupo controle, de nascidos vivos a termo. Cada caso foi pareado com dois controles, de acordo com o sexo e a data de nascimento. Foram realizadas entrevistas com as puérperas e análise de prontuários. Para análise dos dados, foi utilizada regressão logística, seguindo a ordem hierárquica de entrada dos blocos.

**Resultados:**

Participaram 221 nascidos vivos no grupo caso e 442 no grupo controle. Após análise ajustada para os demais fatores em estudo, as maiores chances de prematuridade foram associadas aos primeiros filhos (RC 1,96; IC95% 1,34–2,86; p=0,001), cujas mães possuíam maior renda (RC 2,08; IC95% 1,41–3,08; p<0,001), tiveram filho prematuro prévio (RC 3,98; IC95% 2,04–7,79; p<0,001), sofreram violência durante a gestação (RC 2,50; IC95% 1,31–4,78; p=0,005) e realizaram cesariana (RC 2,35; IC95% 1,63–3,38; p<0,001). Os nascidos vivos de mães que realizaram mais de seis consultas de pré-natal apresentaram menor chance de ser prematuros (RC 0,39; IC95% 0,26–0,58; p<0,001).

**Conclusões:**

Os fatores associados à maior chance de prematuridade foram: maior renda familiar, filho prematuro prévio, primiparidade, violência contra a gestante e cesariana. Ter realizado mais de seis consultas de pré-natal foi associado à menor chance de nascimento prematuro. A violência contra a gestante apresentou associação forte e consistente, permanecendo em todos os modelos finais, devendo servir de alerta para a população e os profissionais.

## INTRODUCTION

Prematurity is considered the main cause of death of children under 5 years of age, especially in the neonatal period.^1,2,3^The risk of morbidity in children born prematurely is also greater due to incomplete fetal development and high susceptibility to infections, which can cause functional disabilities for life.^4^

Each year, more than 15 million premature babies are born in the world.^1-3^This represents approximately 11% of pregnancies in all countries,^2,3^and rates are increasing, including in Brazil, which has a prevalence of 11.5%.^5^

Prematurity, whose etiology is still not well known,^6^is a public health problem^7^because it is multifactorial and because of associated factors such as socioeconomic, demographic, biological, genetic, reproductive, environmental, behavioral, psychosocial conditions, access to health services and service quality, and unidentified causes. Many of these factors are considered preventable,^4,8^which reinforces the importance of prenatal care based on the biopsychosocial concept.^9^

Due to the diversity of the Brazilian population, regionalized population studies that investigate each specific condition must be conducted, taking into account the interrelationship of different factors.^8^The municipality of Governador Valadares, in Minas Gerais, has peculiar characteristics which justify a detailed investigations on this topic due to the worrying increase in prematurity in recent years, as well as the increase in infant mortality and neonatal mortality rates.^10^

In 2010 and 2019, prematurity rates were 6.2 and 9.3 per 100 births, respectively. Infant and neonatal mortality increased from 10.0 to 14.2, and from 7.3 to 8.8 per thousand live births, respectively, in the same years.^10^Of the births in the municipality in 2019, 59.6% were cesarean sections,^10^which corresponds to a very high rate according to the recommendation by the Ministry of Health.

Governador Valadares is among the municipalities with the highest number of cases of violence against women registered in the state, and studies must be conducted to assess the consequence of violence against pregnant women when it comes to prematurity.^11^Another factor to be investigated is the possible impact on duration of pregnancy by the environmental disaster that occurred in Mariana, as it reached the Rio Doce, the only source of supply in the municipality.^12^

This study aimed to verify the association of socioeconomic, demographic, environmental, reproductive, behavioral and health-care factors with prematurity in live births at the Municipal Hospital of Governador Valadares.

## METHOD

Case-control study carried out with live births at the Municipal Hospital of Governador Valadares, from May 2017 to July 2018, whose mothers lived in the municipality or region. This hospital is considered a reference for the cities of Vale do Rio Doce, serves the Unified Health System (SUS) and is the only one in the city and region where there is a Neonatal Intensive Care Unit (NICU).

Premature live births (with gestational age less than 37 weeks) were considered cases. To standardize the definition of gestational age, considering that the most reliable measures were not present in all medical records or prenatal card, the following criterion was adopted: gestational age obtained by examination of ultrasonography and, in the absence of this information, gestational age recorded by the obstetrician followed by that obtained by the date of the last menstruation and, finally, the age defined by the pediatrician.

Controls were selected by pairing with cases, and the following characteristics were required: full term live births (with gestational age equal to or greater than 37 weeks and less than 42 weeks), adequate birth weight (birth weight equal or greater than 2500 g), same sex and same date of birth. Gender matching was necessary because of the greater biological vulnerability of males and the association of males with neonatal mortality.^5,13^In addition, preterm birth occurs more often among boys, accounting for approximately 55% of preterm infants worldwide.^2^Matching with date of birth was aimed at making the sample as similar as possible with regard to perinatal care, including human resources and professional qualification. For each case, two controls were selected.

All live births with any type of congenital malformations, genetic syndromes or lesions of the central or peripheral nervous system diagnosed or suspected at birth were excluded from the study. Cases for which there were no two controls were also not included.

Data was collected through interviews with the mothers, with use of questionnaires with closed questions, even during the hospital stay, within 24 to 48 hours after delivery. Complementary information was obtained on prenatal and medical records of mother and newborn. Data were collected by five researchers previously trained.

**Figure 1:**
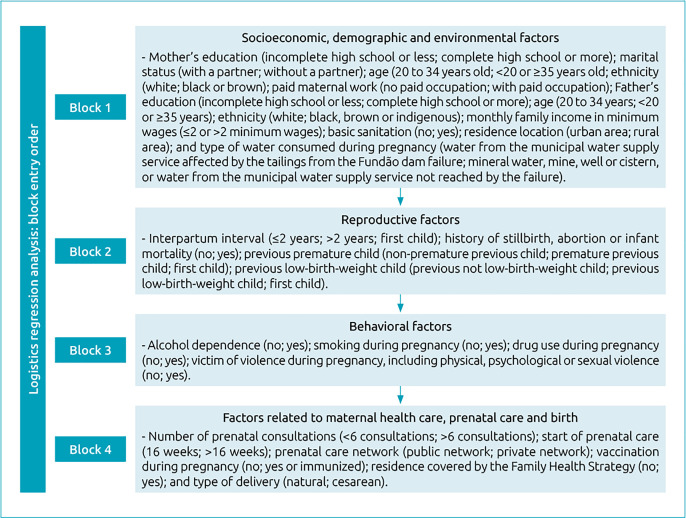
Explanatory model of independent variables divided into blocks and order of entry of factors in the logistic regression analysis.

Prematurity was considered a dependent variable. The independent variables were divided into four blocks:

- block 1) socioeconomic, demographic and environmental factors;
- block 2) reproductive factors;
- block 3) behavioral factors; and block 4) factors related to maternal health care, prenatal care and childbirth.Figure 1 is an explanatory model containing the form of categorization of each variable.

The type of water consumed during pregnancy was evaluated because of the collapse of the Fundão Dam, by the Samarco mining company, which occurred in Mariana in 2015, and reached the waters of the river Rio Doce, the only source that supplies the municipality of Governador Valadares and some neighboring municipalities. The purpose of including this variable was to assess a possible association with prematurity, since water consumed during pregnancy was associated with low birth weight in a study carried out with a similar population.^12^To categorize this variable, it was taken into account that the participants lived in different municipalities, some affected by the tailings from the Fundão dam collapse and others not.

Alcohol intake was detected using the instrument Cutdown, Annoyed, Guilty and Eye-opener,^14^chosen because it is easy to apply, simple and validated for use in Brazil. The categorization of the variables “number of prenatal consultations” and “first prenatal consultation” was defined based on recommendations by the Ministry of Health, which determines the beginning of prenatal care until the sixteenth week of pregnancy and a minimum of six consultations.^15^

The sample size was calculated to allow detecting an odds ratio of 1.8, considering a study power of 80%, significance level of 5% and a relative frequency of 10% of a given exposure factor. Such value was considered because it is a study in which several exposure factors were analyzed.^7,8^The minimum estimated sample size was 213 cases and 426 controls.

Data were archived and analyzed in the Statistical Package for Social Sciences (SPSS) 14.0 program. To verify the associations of independent variables with prematurity, a logistic regression model was used. Associated factors that presented p<0.20 in the bivariate analysis were considered eligible to compose the multivariate models. First, a multivariate analysis of the variables of each block was performed separately, removing those that lost significance. Then, the previously selected variables with p<0.05 were submitted to a new multivariate analysis, following the order of entry of the blocks: first, the variables in block 1, followed by block 2, block 3 and, finally, the variables of block 4, using p<0.05 as a parameter for permanence in the final model.

The project was approved by the Human Research Ethics Committee of the Federal University of Juiz de Fora, on November 28, 2016 (CAAE: 61055716.4.0000.5147). All postpartum women who agreed to participate were informed of the purpose and procedures of the study before signing the free and informed consent form.

## RESULTS

In the period of data collection, according to the Information Technology Department of the Unified Health System,^10^5,141 births were registered in the city, of which 447 live births were premature. In addition to the hospital where the research was conducted, two private hospitals also function as maternity hospitals. At the Municipal Hospital “Governador Valadares”, 332 premature births were identified in the aforementioned period. However, 12 postpartum women were not in the hospital due to possible early discharge, 33 refused to participate in the study, three premature newborns had genetic syndromes and two postpartum women were indigenous and did not speak Portuguese, so it was not possible to interview them. In addition, 61 cases were excluded because we could not find two matching controls. Thus, 221 live births belonging to the case group and 442 to the control group participated in this study.

Male newborns (57.9%) and black or brown-skin newborns were predominant (65.9%). Of the total number of preterms, 173 were considered late preterm (78.3%), 17 moderate preterm (7.7%), 22 very preterm (9.9%) and nine extremely preterm (4.1%).

The socioeconomic, demographic and environmental factors that presented p<0.20 in the bivariate analysis were: mother education (p=0.048), father education (p=0.077), family income (p<0.001) and water consumed during pregnancy (p=0.171) (Table 1).

**Table 1:** Distribution of cases and controls, odds ratio, 95% confidence interval and p-value according to socioeconomic, demographic and environmental factors (Block 1).

	Cases (n=221)		Controls (n=442)		OR	95%CI	p-value
	n	%	n	%			
Mother’s education							0.048
Complete high school or less	101	45.7	238	53.8	Ref.	–	
Complete high school or more	120	54.3	204	46.2	1.39	1.00–1.92	
Mother’s age							0.814
20-34 years	149	67.4	302	68.3	Ref.	–	
<20 or ≥35 years	72	32.6	140	31.7	1.04	0.74–1.47	
Mother’s ethnicity							0.302
White	35	15.8	57	12.9	Ref.	–	
Black, brown	186	84.2	385	87.1	0.79	0.50–1.24	
Marital status							0.273
No partner	182	82.4	348	78.7	Ref.	–	
Partner	39	17.6	94	21.3	0.79	0.52–1.20	
Father’s education**							0.077
Complete high school or less	108	53.7	242	61.3	Ref.	–	
Complete high school or more	93	46.3	153	38.7	1.36	0.97–1.92	
Father’s age**							0.374
20-34 years	138	64.8	295	68.3	Ref.	–	
<20 or ≥35 years	75	35.2	137	31.7	1.17	0.83–1.65	
Father’s ethnicity							0.586
White	48	21.7	88	19.9	Ref.	–	
Black, brown, indigenous	173	78.3	354	80.1	0.90	0.60–1.33	
Family’s monthly income **							<0.001
≤2 minimum wages	138	64.2	326	77.6	Ref.	–	
>2 minimum wages	77	35.8	94	22.4	1.93	1.35–2.78	
Mother’s paid occupation							0.606
No paid occupation	139	62.9	287	64.9	Ref.	–	
Paid occupation	82	37.1	155	35.1	1.09	0.78–1.53	
Sanitation							1.000
No	27	12.2	54	12.2	Ref.	–	
Yes	194	87.8	388	87.8	1.00	0.61–1.64	
Residence							0.330
Urban area	190	86.0	367	83.0	Ref.	–	
Rural area	31	14.0	75	17.0	0.80	0.51–1.26	
Water consumed during pregnancy							0.171
Mineral water, mine, well, cistern or WSS of unaffected municipalities	177	80.1	333	75.3	Ref.	–	
WSS of affected municipalities	44	19.9	109	24.7	0.76	0.51–1.13	

WSS: water supply service; n: number; OR:*Odds Ratio*; 95%CI: 95% confidence interval; Ref.: reference category. **Some mothers were unable or unwilling to inform their partner’s data, such as education (n=67) and age (n=18), in addition to family’s monthly income (n=28), which were considered as absent data in the analysis.

Regarding reproductive factors, the variables previous preterm child (p<0.001) and child with low birth weight (p=0.004) were selected for the multivariate analysis. However, these two variables are highly associated with each other, including a common category (first child), and should not, therefore, be included simultaneously in the regression models. For this reason, only the variable previous premature child was included.

With regard to behavioral factors, only the variable having been a victim of violence during pregnancy (p=0.004) showed a statistically significant association with prematurity (Table 2).

**Table 2:** Distribution of cases and controls, odds ratio, 95% confidence interval and p-value according to reproductive factors (Block 2) and behavioral factors (Block 3).

	Cases (n=221)		Controls (n=442)		OR	95%CI	p-value
	n	%	n	%			
Block 2							
Interpartum interval							0.228
>2 years	81	36.7	166	37.6	Ref.	–	
≤2 years	26	11.8	72	16.3	0.74	0.44–1.25	0.258
First child	114	51.6	204	46.2	1.14	0.81–1.63	0.449
Previous miscarriage							0.465
No	180	81.4	370	83.7	Ref.	–	
Yes	41	18.6	72	16.3	1.17	0.77–1.79	
Previous stillbirth							1.000
No	217	98.2	434	98.2	Ref.	–	
Yes	4	1.8	8	1.8	1.00	0.30–3.36	
Child mortality							1.000
No	216	97.7	432	97.7	Ref.	–	
Yes	5	2.3	10	2.3	1.00	0.34–2.96	
Previous premature child							<0.001
Previous non-premature child	80	36.2	217	49.1	Ref.	–	
Previous premature child	27	12.2	21	4.8	3.49	1.87–6.52	<0.001
First child	114	51.6	204	46.2	1.52	1.08–2.14	0.018
Previous low-birth-weight child							0.007
Previous non-low-birth-weight child	86	38.9	218	49.3	Ref.	–	
Previous low-birth-weight child	21	9.5	20	4.5	2.66	1.37–5.16	0.004
First child	114	51.6	204	46.2	1.42	1.01–1.99	0.044
**Block 3**							
Alcohol addiction							0.449
No	208	94.1	422	95.5	Ref.	–	
Yes	13	5.9	20	4.5	1.32	0.64–2.70	
Smoking							1.000
No	205	92.8	410	92.8	Ref.	–	
Yes	16	7.2	32	7.2	1.00	0.54–1.87	
Drug use							0.314
No	217	98.2	438	99.1	Ref.	–	
Yes	4	1.8	4	0.9	2.02	0.50–8.15	
Victim of violence during pregnancy							0.004
No	195	88.2	418	94.6	Ref.	–	
Yes	26	11.8	24	5.4	2.32	1.30–4.15	

n: number; OR:*Odds Ratio*; 95%CI: 95% confidence interval; Ref.: reference category.

It is important to highlight that 11 postpartum women (1.7%) did not attend any prenatal consultations, eight of them belonging to the case group. In this block, on factors related to maternal health care, prenatal care and childbirth, the following variables presented p value below 0.20: number of prenatal consultations (p<0.001), beginning of prenatal care (p=0.053), prenatal care network (p=0.014), vaccination (p<0.001), residence covered by the Family Health Strategy (p=0.125), and type of delivery (p<0.001) (Table 3).

**Table 3:** Distribution of cases and controls, odds ratio, 95% confidence interval and p-value according to factors related to maternal health care, prenatal care and childbirth (Block 4).

	Cases (n=221)		Controls (n=442)		OR	95%CI	p-value
	n	%	n	%			
Number of prenatal appointments**							<0.001
<6 appointments	79	36.6	90	20.5	Ref.	–	
≥6 appointments	137	63.4	348	79.5	0.45	0.31–0.64	
Start of prenatal care**							0.053
≤16 weeks	186	89.4	364	83.7	Ref.	–	
>16 weeks	22	10.6	71	16.3	0.61	0.36–1.01	
Prenatal care network**							0.014
Public network	166	77.9	376	85.6	Ref.	–	
Private network	47	22.1	63	14.4	1.69	1.11–2.57	
Vaccination during pregnancy							<0.001
No	23	10.4	15	3.4	Ref.	–	
Yes/immunized	198	89.6	427	96.6	0.30	0.15–0.59	
Residence covered by FHS							0.125
No	17	7.7	21	4.8	Ref.	–	
Yes	204	92.3	421	95.2	0.60	0.31–1.16	
Type of delivery							<0.001
Natural	104	47.1	295	66.7	Ref.	–	
Cesarian	117	52.9	147	33.3	2.26	1.62–3.14	

FHS: Family Health Strategy; n: number; OD:*Odds Ratio*; 95%CI: 95% confidence interval; Ref.: reference category. **11 postpartum women did not undergo prenatal care, and these were not included in the analysis of variables beginning of prenatal care and prenatal care network. Nine puerperal women did not remember when the first prenatal consultation was or how many prenatal consultations they had attended, and there were no records in their cards, so it was not possible to include them in the analysis of number of prenatal consultations and beginning of prenatal care.

The variables previously selected by bivariate analysis in each block were put in a logistic regression model. In the analysis of the variables in block 1, monthly family income (p<0.001) maintained a significant association with prematurity and, in block 2, having had a previous premature child (p<0.001). In block 3, the variable that remained in the model was being a victim of violence during pregnancy (p=0.006). In block 4, the variables that maintained a significant association with prematurity were: number of prenatal consultations (p<0.001), beginning of prenatal care (p=0.001), vaccination (p=0.031) and type of delivery (p<0.001).

The significant variables of each block were then submitted to a new multivariate analysis, following the order of entry of the blocks described in Figure 1. The result of the logistic regression is shown in Table 4.

**Table 4:** Final result of the logistic regression model including factors associated with prematurity.

Blocks		OR	95%CI	p-value
Block 1	Monthly family income			<0.001
	≤2 minimum wages	Ref.	–	
	>2 minimum wages	2.08	1.41–3.08	
Block 2	Previous premature child			<0.001
	Previous non-premature child	Ref.	–	
	Previous premature child	3.98	2.04–7.79	<0.001
	First child	1.96	1.34–2.86	0.001
Block 3	Victim of violence during pregnancy			0.005
	No	Ref.	–	
	Yes	2.50	1.31–4.78	
Block 4	Number of prenatal appointments			<0.001
	<6 appointments	Ref.	–	
	≥6 appointments	0.39	0.26–0.58	
	Type of delivery			<0.001
	Natural	Ref.	–	
	Cesarian	2.35	1.63–3.38	

OR: *Odds Ratio*; 95%CI: 95% confidence interval; p-value: level of statistical significance; Ref.: reference category.

## DISCUSSION

The results of this study reinforce the need for a broad biopsychosocial and multidisciplinary approach to pregnant women, with investigations during prenatal consultations that go beyond vital signs, clinical and pharmacological examinations, investigating each specific condition without losing sight of the interrelationship of different factors.^16^Some factors that were associated with prematurity are considered preventable.

Early detection of violence against pregnant women can favor the course of a healthy pregnancy. Studies referring to the association of prematurity with violence against pregnant women are gaining more and more prominence, but there are still few Brazilian studies that assess the consequences of this factor for maternal and child health and its relationship with preterm birth.^6^The exposure of pregnant women to violence significantly increases the chance of premature birth (OR 1.91; 95%CI 1.60–2.29)17 (OR 1.46; 95%CI 1.27–1.67),^18^as observed in two systematic reviews and meta-analyses that included studies conducted in different countries on the relationship between violence during pregnancy and perinatal outcomes.^17,18^

Our findings should serve as a warning to the population and health professionals, since, in Brazil, the rate of violence against women has increased in recent years,^19^with a high prevalence of cases among pregnant women.^20^Violence against pregnant women is associated to factors such as alcohol dependence, lack of prenatal care, emergency use of health services and diseases during pregnancy, such as gestational diabetes, HIV and syphilis.^21^Effective programs to identify victims of violence and allow intervention, even during pregnancy, are fundamental.^18,21^

Live births to mothers with a family income above two minimum wages were more likely to be premature, unlike what has been reported in other studies, which associate premature birth with greater vulnerability of low-income women, due to unfavorable health conditions, low education and less access to and use of health services.^16,22^The participants in this study were predominantly of low economic status, with a monthly income of less than two minimum wages (70.0%), who performed prenatal care exclusively through SUS (81.7%) and were all users of the same public hospital, so the sample was homogeneous between cases and controls in terms of socioeconomic characteristics and access to health services. The results of this study show, in a way not commonly observed, that prematurity can also be associated with better economic conditions.

A study carried out in New Zealand, aiming to assess changes in the incidence of preterm birth over 20 years, found that prematurity rates increased by 71.9% in the population living in the wealthiest areas and only 3.5% in the poorest areas, challenging traditional thinking about the association of socioeconomic factors with premature birth.^23^

In a study with a birth cohort in Pelotas (RS), cesarean section was more frequent among richer and more educated mothers,^24^which also helps to reinforce the hypothesis of a greater chance of premature birth in mothers with higher incomes, since this group also had more postpartum women who underwent cesarean. In our study, postpartum women who underwent cesarean were 2.3 times more likely to have a premature child, compared to those who had natural birth. Part of this greater chance can be explained by the fact that some pregnancies were interrupted due to obstetric or fetal indications, so the analysis of this result requires caution.

According to Leal et al.,^25^Brazil has the highest rates of cesarean sections in the world, occurring in 55.0% of pregnant women, with continued growth in recent years, including even the lowest-income groups. Premature births can be partly attributable to unnecessary cesarean sections, causing iatrogenic prematurity,^4,6^which makes it essential to invest in actions aimed at preventing this type of avoidable prematurity related to undue termination of pregnancy, such as cesarean sections without technical indication.^5^

The first children and those whose mothers had a premature previous child had a greater chance of prematurity. The biological mechanism related with how parturition can influence the occurrence of preterm birth is not well defined,^26^but studies show a significant association of these factors.^16,27^The effects of parturition in subsequent pregnancies are affected by the perinatal outcomes of previous pregnancies, with the risk of premature birth when the previous child was also born prematurely.^26^

A cohort study carried out in Japan identified that one in six women with a previous preterm child had recurrent preterm birth.^28^Women who had a preterm child in their first pregnancy have an increased risk of recurrence, being 14 times higher when the birth occurs at a gestational age below 34 weeks.^29^Premature birth in a previous pregnancy can be considered a predisposition marker for other subsequent adverse outcomes, and these factors should be given more attention in prenatal consultations.

It should be noted that the variable “previous low-birth-weight child” was excluded from the final regression model due to the collinearity with the variable “previous premature child”. As these variables are highly associated and when performing the analysis one can be replaced by the other, the values of OR, 95%CI and p value were practically the same and the significance of all was maintained. Thus, it is important to highlight the importance of professionals also paying attention to the greater chance of a premature birth in women who had a low-birth-weight child in a previous pregnancy. Having had six appointments or more was associated with a lower chance of preterm birth, as reported in other studies.^7,16,30^In Minas Gerais, prematurity was three times higher among women who had less than six prenatal appointments compared to those who had seven or more (OR 3.43; 95%CI 2.96–3.98; p<0.001).^30^The case group had, on average, fewer consultations than the control group; therefore, the association of prematurity with the number of prenatal consultations should be viewed with caution and requires specific studies due to a possible problem of reverse causality. Was prematurity due to the smaller number of appointments or was the number of appointments due to lower gestational age? The exclusion of this variable from the regression models did not significantly change the association of the other variables with prematurity (results not shown).

This study has the recall bias of postpartum women and the failure or absence of some records in the pregnant woman’s card and medical records as limitation, in addition to the sample being consecutive, from a single service. Another limitation was not using any instrument to assess violence against women besides direct questions being asked to mothers. The use of a specific instrument could detect a greater number of mothers who suffered violence during pregnancy. Despite the limitation, this factor was significantly associated with prematurity.

We conclude that the factors associated with a greater chance of prematurity were: higher family income, previous preterm child, primiparity, violence against pregnant women, and cesarean section. Having attended more than six prenatal consultations was associated with a lower chance of preterm birth. Violence during pregnancy showed a strong and consistent association, as it remained in all final models, with a high odds ratio compared to the various factors studied, and should serve as a warning for both the population and health professionals.

This research was important to raise awareness of health professionals and managers about the main determinants of prematurity affecting the region of Governador Valadares, in order to adapt gestational care for the detection and prevention of health problems and reduce prematurity and neonatal morbidity and mortality.
